# And yet another transcription factor—the complex world of fruit ripening

**DOI:** 10.1093/plphys/kiad673

**Published:** 2024-01-04

**Authors:** Lara Pereira

**Affiliations:** Assistant Features Editor, Plant Physiology, American Society of Plant Biologists; Ecology and Evolutionary Biology, School of Biosciences, University of Sheffield, Sheffield S10 2TN, UK

Fruit ripening is a complex process undergone by mature fruits to achieve successful seed dispersal. After ripening initiation, fruits change their color, their flesh becomes soft, and a multitude of organic compounds are synthesized to achieve delicious flavor and aroma. Although the ultimate evolutionary aim of fruit ripening—seed dispersal—might seem obsolete in modern societies, fruit ripening remains one of the most relevant physiological processes in plants. Fruits are an essential part of human diet and a main source of vitamins, and ripening determines nutritional quality, flavor, and post-harvest life. Therefore, understanding and modulating ripening is key for breeders and food industries.

Tomato has been established as a model organism to study climacteric fruit ripening; these are the fruits in which the onset of ripening is marked by a peak of respiration, followed by an autocatalytic synthesis of the plant hormone ethylene. Epigenetic changes, hormone crosstalk, and an intricate network of transcription factors all contribute to a tight and elaborate regulation (Brumos 2021). A few classical mutant lines, such as *non-ripening* (*nor*), *ripening inhibitor* (*rin*), and *Colorless non ripening* (*Cnr*), displayed a delayed or even absent ripening phenotype. The transcription factors underlying these mutants are traditionally described as “master regulators” (Giovannoni et al. 2017). However, CRISPR-Cas9 and advanced genomic technologies allowed to re-evaluate the role of these transcription factors; the drastic nonripening phenotypes observed in the mutants were due to underlying gain-of-function mutations (Nakano et al. 2012; Brumos 2021). When knockout mutants were characterized, they presented mild effects in ripening (Wang et al. 2019). In addition, during the last 2 decades, many other transcription factors have been linked to ripening and characterized as positive or negative regulators, nourishing the hypothesis of the existence of a quantitative, tangled genetic network to control fruit ripening (Brumos 2021). And yet, some main players are yet to be identified.

In the current issue of *Plant Physiology*, Jiang et al. (2023) characterized SlWOX13, a WUSCHEL-related homeobox (WOX) transcription factor, as a novel positive regulator of fruit ripening in tomato, using an approach that combines molecular biology and multi-omics. SlWOX13 presents a ripening-specific spatiotemporal expression pattern, and its expression increases and decreases when applying ethylene and ethylene inhibitors, respectively, suggesting a role in fruit ripening. The slwox13 mutants generated by CRISPR-Cas9 showed delayed repining phenotypes, whereas SIWOX13 overexpression transgenic lines ripened earlier, indicating that SlWOX13 is an enhancer of fruit ripening.

Comparative transcriptomics and ChIP-seq analyses revealed that, among the 5,743 ripening-associated genes regulated by SlWOX13, 422 were potential direct targets. These putative SlWOX13-targeted ripening-related genes were involved in interesting physiological processes such as ethylene biosynthesis, hydrolase activity, and hormone activity. Furthermore, the authors showed that SlWOX-13 binds and activates the promoters of the key ripening transcription factors RIN, NOR, and NAC4.

An interesting question not yet fully answered is related to the developmental competence the fruit must acquire to ripen. In climacteric fruits, the response to ethylene is different depending on whether the fruit is developmentally ready to ripen—the landmark is usually viable, mature seeds. If exogenous ethylene is applied to a young, immature fruit, ripening is not promoted; however, when the fruit tissues are competent, exogenous ethylene will initiate ripening. How this transition happens is not yet fully understood, and most of the players at the molecular level remain unknown. Demethylation of promoter regions by DEMETER-like DNA demethylase 2 (DML2) seems to be essential and at least partially responsible for this developmental change (Liu et al. 2015). Other epigenetic modifications such as histone demethylation and acetylation also affect ripening initiation (Chen et al. 2020). Jiang et al. (2023) hypothesized that SlWOX13 is involved in the feedback loop that leads to the autocatalytic ethylene peak by directly interacting with relevant transcription factors such as RIN and NOR and by altering fruit epigenetics through DML2 regulation. Other characterized transcription factors such as RIN, NOR, and MADS1 also activate genes belonging to the ethylene biosynthesis and regulation pathway, cell wall–related enzymes, and the carotenoid pathway (Fujisawa et al. 2013; Yuan et al. 2016; Liu et al. 2021), indicating that the network is highly redundant and a myriad of genes contribute to trigger and amplify ripening signals.

In summary, the regulator SlWOX13 is involved in modulating the transcription factor cascade that promotes ripening and carotenoid synthesis (Fig. 1). Moreover, SlWOX13 affects DML2 expression levels, and therefore it could have a role in the epigenetic changes that boost the competence to ripe. Although the gene network controlling fruit ripening is complex, all newly identified players open an additional avenue to explore a strategy that would convince breeders and consumers alike—tasty, aromatic fruits that ripen slowly, conserve a good texture for longer, and present an increased post-harvest life.

**Figure 1.**
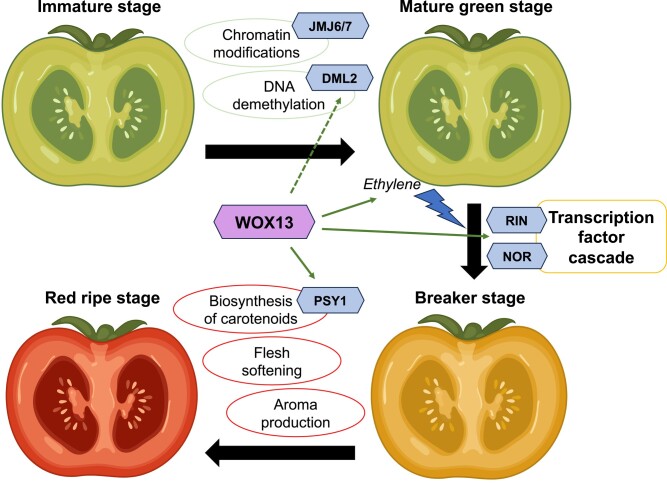
Summary of the role of SlWOX13 in tomato fruit ripening. The transcription factor SlWOX13 is highlighted in the center of the schematic, and other genes are represented by hexagons. Molecular and physiological changes are circled.

## Dive Curated Terms

The following phenotypic, genotypic, and functional terms are of significance to the work described in this paper:

DML2 Gramene: AT3G10010
DML2 Araport: AT3G10010

MADS1 Gramene: GRMZM2G171365
MADS1 Araport: GRMZM2G171365

fruit AmiGo: PO:0009001
